# Theoretical Study of the ESIPT Process for a New Natural Product Quercetin

**DOI:** 10.1038/srep32152

**Published:** 2016-08-30

**Authors:** Yunfan Yang, Jinfeng Zhao, Yongqing Li

**Affiliations:** 1 Department of Physics, Liaoning University, Shenyang 110036, P. R. China; 2 State Key Laboratory of Molecular Reaction Dynamics, Dalian Institute of Chemical Physics, Chinese Academy of Sciences, Dalian 116023, P. R. China

## Abstract

The investigation of excited-state intramolecular proton transfer (ESIPT) has been carried out via the density functional theory (DFT) and the time-dependent density functional theory (TDDFT) method for natural product quercetin in dichloromethane (DCM) solvent. For distinguishing different types of intramolecular interaction, the reduced density gradient (RDG) function also has been used. In this study, we have clearly clarified the viewpoint that two kinds of tautomeric forms (K_1_, K_2_)originated from ESIPT processconsist inthe first electronic excited state (S_1_). The phenomenon of hydrogen bonding interaction strengtheninghas been proved by comparing the changes of infrared (IR) vibrational spectra and bond parameters of the hydrogen bonding groups in the ground state with that in the first excited state. The frontier molecular orbitals (MOs)provided visual electron density redistribution have further verified the hydrogen bond strengthening mechanism. It should be noted that the ESIPT process of the K_2_ form is easier to occur than that of the K_1_ form via observing the potential energy profiles. Furthermore, the RDG isosurfaces has indicated that hydrogen bonding interaction of the K_2_ form is stronger than that of the K_1_ formin the S_1_ state, which is also the reason why the ESIPT process of the K_2_ form is easier to occur.

The ESIPT process resulted from photo-protolytic phenomena is one of the most important processes in photochemistry, photobiology and so forth^1^,^2^,^3^. The hydrogen bonding interaction exists in numbers of organic compounds, which possess hydrogen donor group and hydrogen acceptor group. Upon the photo-induced process, the hydrogen bonding interaction could be fast impacted, the primary properties of the compounds could be changed concomitantly. The intermolecular hydrogen bondbetween solvent and solute moleculescan be strengthened dramaticallyin the excited states, which has been proposed by Han and co-workers^4^,^5^,^6^,^7^,^8^,^9^,^10^,^11^. The ESIPT process has been investigated extensively by various theoretical and experimental measures since the phenomenon was experimentally first observed by Weller *et al.* in 1955^12^. In fact, the ESIPT reaction is an ultrafast process occurred in the femto- to picosecond time scale, where the protontransfer pathway is linked by a hydrogen bond, and the proton donor group and acceptor groupin close proximity^13^.

The hydrogen bonding interaction could offer the driving force for the ESIPT process^14^,^15^. To date, numbers of scientists have widely studied some compounds that can form one or more intramolecular hydrogen bonds. In most cases, the hydrogen bond group is composed of a proton donor and acceptor. For example, Pi-Tai Chou *et al.* has reported that the ESIPT processes of the 3-hydroxyflavone (3HF) monomer and the 5-hydroxyflavone (5HF) monomer, respectively^16^. In a similar way, Jin-Feng Zhao *et al.* reported that two ESIPT processes exist in D3HF molecule, the conclusion has been demonstratedthat the excited-state double protons transfer (ESDPT) process cannot occur simultaneously along with corresponding hydrogen bonding pathway^17^. However, we have paid great attention to the peculiarconstruction ofnew natural product quercetin. It is noteworthy that thereare two intramolecular hydrogen bonds sharedacommon proton acceptorin thequercetin. It is puzzling that the two ESIPT processes exist in the quercetin molecule which one should take place first? However, the novel phenomenon is hardly illustrated experimentally. Therefore, we will give people visualized insight into the particular ESIPT processes by means of the detailed theoretical calculation in this study. As shown in the Fig. 1, the configuration of quercetinmolecule isso stable in the ground state (S_0_) that the ESIPT processes cannot spontaneously occur. Upon the photo-induced process, the new tautomer forms (K^S1^_1_, K^S1^_2_) can be generated by means of the fast ESIPT processes in the S_1_ state. The compounds K^S1^_1_and K^S1^_2_ will play an important role in the most application fieldsand will have wide application prospects, for example, the filters materials, thefluorescence sensors, the laser dyes and LEDs, *etc*.^18^,^19^,^20^,^21^,^22^,^23^,^24^,^25^,^26^,^27^.

The natural product quercetin, a good molecular construction system, is one of the most extensivelysubsistent flavonoids. It possessesthe extensively biological activities, in especial the natural product has been used as food supplement such asit has been reported in some documents that the quercetin has many therapeuticfunctions in food supplement. For example, the anticancer, the antiviral, anti-inflammatory and anti-neoplasticfunction^28^,^29^,^30^,^31^. The two hydrogen bond groups existed in the quercetinstructureconsist of a common proton acceptor and twoproton donors in close proximity. The both proton donorsare thehydroxyl groupwhile the common proton acceptor is the carbonyl oxygen atom in the hydrogen bondgroup moiety^32^. In addition, two hydrogen bond groups form the five-membered and six-membered ring structure, respectively. Two ESIPT processes can occuralong with the orientation of corresponding hydrogen bond group in the ring structure^33^,^34^. Therefore, the interactions of hydrogenbond have been defined as the important driving force in the ESIPT process^35^,^36^. Simkovitch *et al.* have investigated the time-resolvedfluorescence of the quercetin experimentally throughthe steady-state absorption spectroscopy and fluorescent up-conversiontechniques^32^. However, the above measures cannotprimelyaccount for the two ESIPT processes that the proton jumps from the corresponding proton donor to the common proton acceptor. Herein, to comprehend the two ESIPT processes occurredon the corresponding hydrogenbond group, we have theoretically investigated the ESIPT processes in terms of the quantum chemical calculation methods.

## Results and Discussion

**Figure 2:**
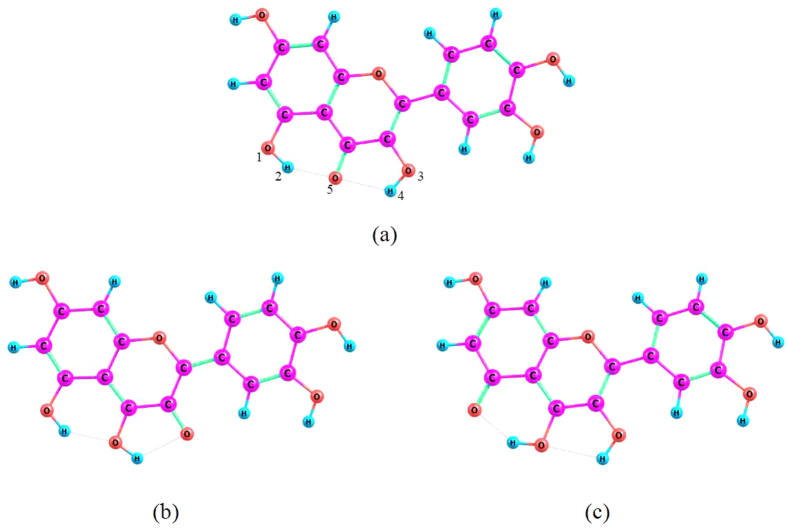
Optimized geometrical configurations of the quercetin molecule: the normal form (**a**), The tautomeric K_1_ form (**b**), The tautomeric K_2_ form (**c**). The blue: H, the pink: C, the red: O. The dash line refers to the intramolecular hydrogen bond. (For interpretation of the references to color in this figure legend, the reader is referred to the web version of this article).

The quercetin has the normal configuration in the S_0_ state. On the contrary, upon the photo-inducedprocess there are two tautomeric forms K_1_ and K_2_ resulted from ESIPT processes in the S_1_ state. These structures have been fully optimized by DFT method in the S_0_ state and TDDFT method in the S_1_ state. Herein, the normal structure as well as the K_1_ and K_2_ formslocated in S_1_ state have been shown in Fig. 2(a–c), respectively. It should be noted that the intramolecular hydrogen bonds have existed initially in the S_0_ state, in which the constructions of the five-membered and the six-membered ring are linked by the each intramolecular hydrogen bond group. In order to illustratepreferably the all above phenomena, we will come up with a few accessibleevidences that cannot be provided experimentally.

### The optimization of configurations

The natural product quercetin has been optimized by means of the DFT/TDDFT methods throughout based on B1B95 function as well as 6–31++G (d, p) basis set in the DCM solvent. The three structures have been optimized and presented on Fig. 2. It is evident that the four intramolecular hydrogen bond groups (O_1_-H_2_···O_5_), (O_3_-H_4_···O_5_), (O_5_-H_2_···O_1_) and (O_5_-H_4_···O_3_) can be observed from the three planar geometric structures. The primary bond lengths (Ǻ) and bond angles (°) relevant to the hydrogen bond groups have been listed in Table 1. The bond lengths O_1_-H_2_ and O_3_-H_4_ of normal form are optimized to be 0.986 Ǻ and 0.976 Ǻ in S_0_ state, but they drastically increase to be 1.001 Ǻ and 0.983 Ǻ in the S_1_ state, respectively. Meanwhile, wehave observed that the bond lengths O_5_-H_2_ and O_5_-H_4_ obviously convert from 1.746 Ǻ and 2.022 Ǻ in the S_0_ state to 1.663 Ǻ and 1.984 Ǻ in the S_1_ state, respectively. The hydrogen bond angles O_1_-H_2_···O_5_ and O_3_-H_4_···O_5_ are enlarged respectively from 148.2° and 117.9° to 152.6° and 120.1° upon photo-excitation process. Therefore, we can make a conclusionthat the intramolecular hydrogen bonds O_1_-H_2_···O_5_ and O_3_-H_4_···O_5_ are strengthened in the S_1_ state.

The fast ESIPT processes occurred in the S_1_ statehave resultedin the distinct changes of the molecular structure, in which the new hydrogenbonds(O_5_-H_4_···O_3_), (O_5_-H_2_···O_1_) have been constituted in tautomeric forms K_1_ and K_2_, respectively. It is very interesting that theO_3_-H_4_ bond length obviouslyreduced from 2.013 Ǻ in the S_1_ state to 1.809 Ǻ in S_0_ state, while the bond length of O_5_-H_4_ increased from 0.979 Ǻ in the S_1_ state to 1.000 Ǻ in the S_0_ state forthe hydrogen bond O_5_-H_4_···O_3_ of K_1_ form. The above analysis results have indicated that hydrogen bond O_5_-H_4_…O_3_ is stronger in the S_0_ state than that in the S_1_ state. In addition, the phenomenon of the intramolecular hydrogen bond strengthening has also been verified by the change of O_5_-H_4_...O_3_ bond angle, whichchanges from 116.9° in the S_1_ state to 124.0° in the S_0_ state.

### Calculated spectrum of absorption and emission

**Figure 3:**
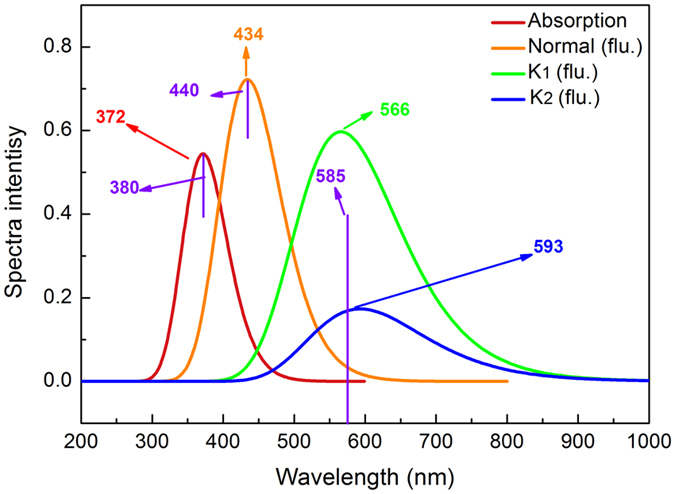
Theoreticalsimulating the absorption and emissionspectrum of the quercetin molecule. The violet vertical lines stand for the corresponding peak values in the experimental. The detail explanations of curves can be given by the legend on the top right corner.

The UV-vis spectra ofquercetin has been investigated experimentally via steady-state measuring method by the researchers Simkovitch *et al.*, and the information of absorption and emission spectra have been revealed in their paper. However, the mechanism of the ESIPT processes is very elusive in the quercetin molecule^32^. Therefore, for further gaining the fluorescent emission and absorptionspectrum, wehave carriedout the theoretical calculation based on the quantum chemistry methods. For comparison to experiment, the spectrum has been displayed in Fig. 3. As shown in the Fig. 3, the top-right legend has clearly illustrated the significance of each spectral line. In addition, the violet vertical lines stand for corresponding peak values obtained in the experiment. It has been found that the absorption peak assigned to the S_0_ → S_1_ transition process locates in 372 nm, which hasan amazingcoincidencethat the absorption wavelength isabout 380 nm in the experiment^32^. Following the photo-excitation process, quercein molecule will go through a fast radiative decay process from the S_1_ state to the S_0_ state, the fluorescence emission peak of normal construction at 434 nm is extremely close to the experimental value of 430 nm. Besides, the emission peaks of K_1_ and K_2_ forms are located at 566 nm and 593 nm, which are alsocoincident with the experimental peak value of 585 nm. It should be noted that the large Stokes shift values are 194 nm and 221 nm between the absorption peak and the emission peaks of tautomeric forms (K_1_, K_2_), respectively, which have been observed in the spectral graph. The large Stokes shifts have suggestedthat the tautomeric structures have drastic changes, which compare with the normal structure. The unusual changes of photophysical property are frequently accompanied by the enormously changes ofmolecular structure, such as the ESIPT processes^37^. Therefore, we draw a conclusion that the ESIPT processes can take place along with the orientation of intramolecular hydrogen bonds in the five-membered ring and the six-membered ring, sinceboth hydrogen bondinginteractionsare strengthened following the photo-excitation.

### Infrared (IR) vibrational spectra analysis

**Figure 4:**
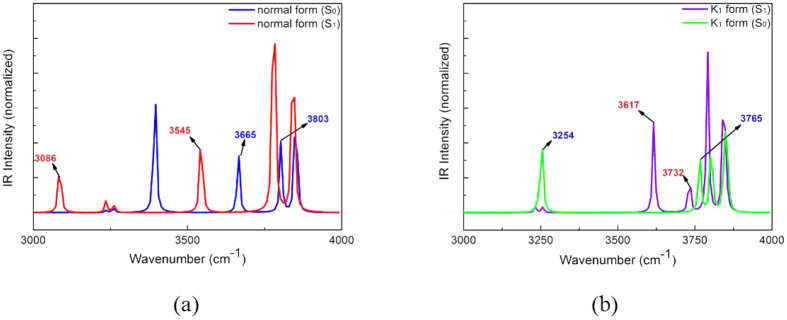
The calculated IR vibrational spectra of thehydrogen bond groups O_1_-H_2_, O_3_-H_4_ and O_5_-H_4_ stretching absorption band in the S_0_ and S_1_ state. The IR vibrational spectra of the normal form (**a**) The IR vibrational spectra of the tautomericK_1_ form (**b**) The legend can give reader the detail explanations.

The infrared (IR) vibrational spectrumis one of the mostprime tools for investigating thehydrogenbond strengthening^38^. Therefore, the effect of the hydrogenbond strengthening can be further illustrated by comparing the IR vibrational spectra of fluorophore in the S_0_ with that in the S_1_ state. The quantum chemical calculation has been carried out for obtaining the IR vibrational spectra of different electronic states. In Fig. 4(a), the IR vibrational spectra of hydroxyl groups O_1_-H_2_ and O_3_-H_4_ have been shown. It is so easy to find that the O_1_-H_2_ stretching vibrational frequency has a distinct red-shifted 579 cm^−1^ from 3665 cm^−1^ to 3086 cm^−1^ in the S_0_ → S_1_ state. Analogously, the O_3_-H_4_ stretching vibrational frequency has a relatively minor red-shifted 258 cm^−1^ from 3803 cm^−1^ to 3545 cm^−1^. Therefore, these analyses haveshed light onthe viewpoint that two hydrogen bonds (O_1_-H_2_···O_5_), (O_3_-H_4_···O_5_) have been obviously enhanced in the S_1_ state. Moreover, IR vibrational spectra of hydrogen bond groups on K_1_ form have been shown in Fig. 4(b). It should be noted that hydroxyl group O_1_-H_2_ stretching vibrational frequency has a slight red-shifted 33 cm^−1^ from 3765 cm^−1^ to 3732 cm^−1^ in the S_0_ → S_1_ state. However, hydroxyl group O_5_-H_4_ stretching vibrational frequency exists a distinct blue-shifted 363 cm^−1^ from 3254 cm^−1^ to 3617 cm^−1^ in the S_0_ → S_1_ state. The results can indicate that the hydrogen bond O_5_-H_4_···O_3_ has been obviously strengthened in the S_0_ state. In Fig. 4(b), the IR vibrationalspectra of the K_2_ form haven’t been shown, since the K_2_ form is nonexistent in the S_0_ state. This is also the reason why we cannot compare the IR vibrational spectra of the hydrogen bond group in theS_1_ state with that in the S_0_ state for the K_2_ form.

### Frontier molecular orbitals (MOs) analysis

**Figure 5:**
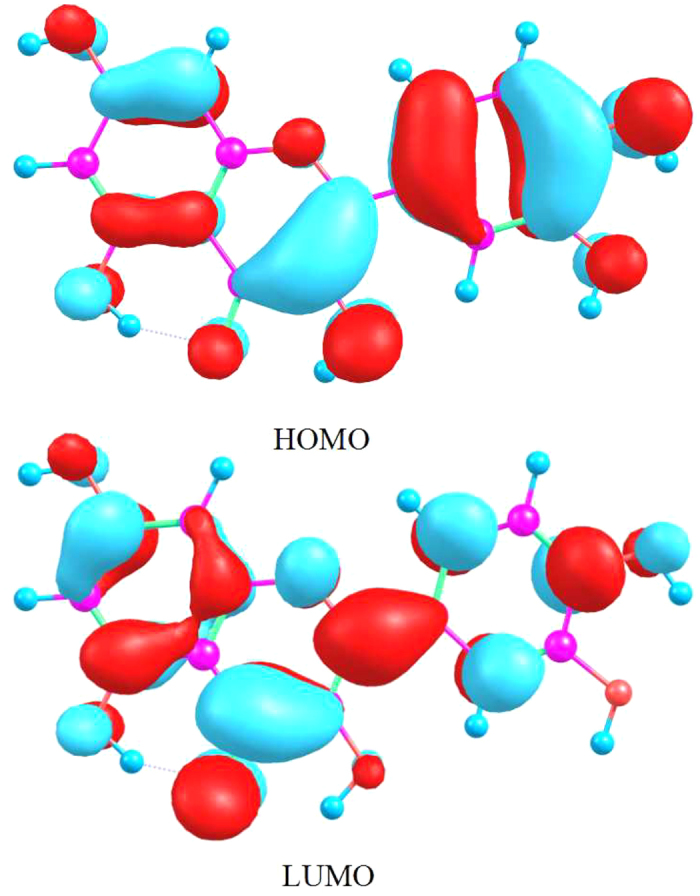
The visual electron population of the frontier molecular orbitals HOMO and LUMO.

To the best of our knowledge, upon photo-induced processthe electron population in the quercetin molecule will be significantly changed. Herein, the frontier MOs theory has been applied to comprehend the properties of the electronic excitation. The electron cloud around the molecule is subdivided into different molecular orbitals possessed of different energy levels, where the highest occupied molecular orbital (HOMO) and the lowest unoccupied molecular orbital (LUMO) tremendously affect the reaction in this study. The HOMO with π character and the LUMO with π^*^ character have been shown in Fig. 5. The typical ππ* character has been primarily assigned tothetransition from the HOMO to LUMO, and the transitioncomposition of the HOMO → LUMO and oscillator strength of the first excited state have been shown in Table 2. It is evident that the electron density of hydroxyl oxygen O_1_ and O_3_ has decreased in the LUMO, which induces the dissociation of hydrogen protons. Meanwhile, the electron density of ketonic oxygen O_5_ has increased distinctly, which contributes to attracting the hydrogen protons dissociated from hydroxyl group. In addition, we have described the change of atomic charge for the hydrogen bond groups via Mulliken charge population analysis. Herein, we found that the negative charge of atom O_1_ and O_3_ has decreased from −0.562 and −0.582 in the S_0_ state to −0.557 and −0.571 in the S_1_ state, respectively. On the contrary, the negative charge of atom O_5_ increases from −0.698 to −0.706. In conclusion, the redistribution of electron population can strengthen the intramolecular hydrogen bond (O_1_-H_2_···O_5_) and (O_3_-H_4_···O_5_) upon photo-induced process, which further contributes to the proceeding of ESIPT processes.

### The potential energy profiles analysis

**Figure 6:**
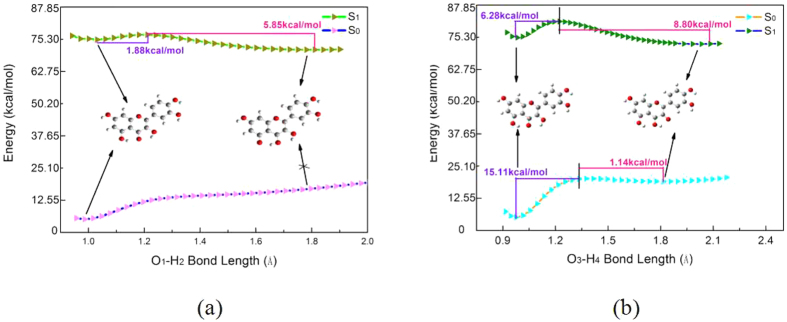
The function curves of the corresponding energy versus the O_1_-H_2_ bond length (**a**) The corresponding energy versus the O_3_-O_4_ bond length (**b**) The numerical values in the graphs stand for the potential barriers of the proton transfer process.

To further explain the ESIPT processes of the quercetin, we have plotted the potential energy curves of the reaction pathways that the protons migrate from the hydroxyl groups to the carbonyl group. The potential energy curves are the function of corresponding energy versus O-H bond lengths, as shown in Fig. 6. The energy of corresponding molecular structurein S_0_ state or S_1_ state have been calculated with the hydroxyl group (O-H) bond lengths increased by the fixed step sizes. Although the TDDFT method is unlikely to accurately acquire the correct order of closely spaced excited states, a number of foregoingresearch work have manifested that the method was relatively reliable with respect to analyzing qualitatively the reaction pathways and the potential barrier of ESIPT processes^39^. The potential energy curves of six-membered ring proton transfer process shown in Fig. 6(a) have revealed that the energy of structure is graduallyincrease with augment of bond length O_1_-H_2_, where the energyhas not showna sign of slowing in the S_0_ state. Therefore, in this case, the proton transfer process cannot occur in the S_0_ state. This phenomenon has definitelyillustrated that the K_2_ form was nonexistent in the S_0_ state. On the contrary, upon photo-induced process, it is clearly observed from the figure that the potential barrier 1.88 kcal/mol of ESIPT process is almost negligible, the ESIPT process iscomparatively easy to occur in the S_1_ state. However, as shown in Fig. 6(b), for the five-membered ring segment of the quercetin molecule, the potential barrier 6. 28 kcal/mol of ESIPT process in the S_1_ state has indicated that ESIPT process is more difficult to occur than the six-membered ring proton transfer process. It should be noted that a large potential barrier (15. 11 kcal/mol) in the S_0_ state has been exhibited in the Fig. 6(b), which indicates the proton transfer process cannot occur spontaneously in the S_0_ state. In addition, the potential barrier of reversed proton transfer is 8.80 kcal/mol in the S_1_ state and is 1.14 kcal/mol in the S_0_ state. Further, we could make a conclusion that the reversed proton transfer process of K_1_ formis easier to occur in the ground state than that in the first excited state. Herein, we have known that the hydrogen bond O_5_-H_4_···O_3_ of the K_1_ form is stronger in the ground state. So the reversed proton transfer process can be enhanced by the hydrogen bonding interaction.

### Discriminating weak interaction types by filling color to RDG isosurfaces

**Figure 7:**
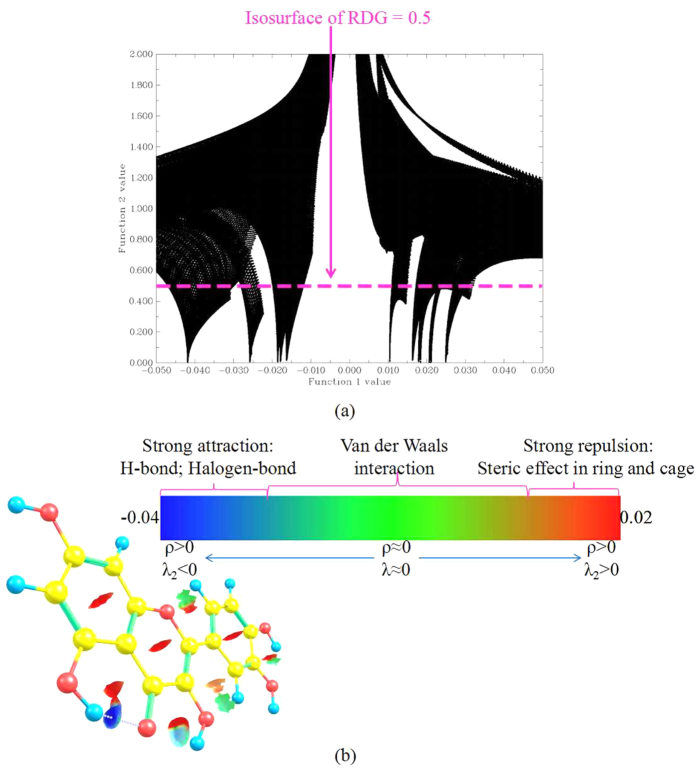
Scatter plot of the reduced density gradient (RDG(r)) versus Ω(r) are expressed as Function value 1 and Function value 2, respectively (**a**) The visual diagram of RDG isosurfaces (**b**) The color gradient corresponding to the diverse types of the interaction have been shown in the figure legend.

For distinguishing different types of interaction, herein the RDG function has been used^40^. The equation can be expressed as


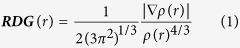


where ρ (r) is the total electron density, the RDG (r) is the reduced density gradient of the exchange contribution. According to Bader’s Atoms in Molecules (AIM) theory^41^, the relative to the second largest eigenvalue λ_2_ of Hessian matrix of electron density and the total electron density ρ (r) may be written in the form


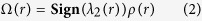


where the weak interaction depends not only on the electron density ρ, but also is concerned with the eigenvalue λ_2_. Herein, we have utilized λ_2_ to distinguish the types of the bonding (λ_2_ > 0) and antibonding (λ_2_ < 0) interaction. Therefore, the sign λ_2_ has been further analyzed via plotting the scatter diagram of the function 1 (RDG) value versus the function 2(Ω(r)) value. As shown in Fig. 7(a), in orderto clearly describe the different types of the interactions, we have usedthe color gradient to stand for ρ (r) and λ_2_ value, and filledinthe RDG isosurfaces. As shown in Fig. 7(b), the visual graph can be obtained via the visual software Chemcraft. The contour value is set as 0.5, the values range of RDG isosurfaces is set as −0.04 to 0.02. From the Fig. 7(b), it could be greatly noted that thehydrogen bonding interaction of the six-membered ring is stronger than that of the five-membered ring in the S_1_ state. Further, the ESIPT process in six-membered ring segment is easier to occur than that in five-membered ring segment.

## Conclusion

In summary, on the basis of DFT/TDDFT methods, the viewpoint that tautomeric forms (K_1_, K_2_) originate from the ESIPT processes has been successfully proved by analyzing the fluorescent spectroscopy and potential energy curves. We have made a importantconclusion that the intramolecular hydrogen bonding interaction can be enhanced in the S_1_ state via comparing the changes of bond parameters of hydrogen bond groups in the S_0_ state with that in the S_1_ state. In addition, the frontier MOs analysishas also confirmed the hydrogenbondinginteraction strengthening upon the process of photo-excitation. However, it’s worth noting thatthe hydrogen bond O_5_-H_4_…O_3_ of K_1_ form has become stronger in the S_0_ state than that in the S_1_ state. Therefore, the reversed proton transfer process can be greatly facilitated by the stronger hydrogenbonding interaction in the S_0_ state. On the Fig. 6(b), we have found that the potential barrier of the reversed proton transfer process is 8.80 kcal/mol in the S_1_ state and is 1.14 kcal/mol in the S_0_ state. We have made a conclusion that the reversed proton transfer process of the K_1_ formis easier to occur in the ground state than that in the first excited state. However, for the ESIPT process, the potential barrier 1.88 kcal/mol of K_2_ form is almost nonexistent. On the contrary, the potential barrier of K_1_ form is 6.28 kcal/mol, so the ESIPT process of K_2_ form is easier to occur than the process of K_1_ form. Besides, on the Fig. 7, the RDG isosurfaces have clearly indicated that the interaction of hydrogen bond (O_1_-H_2_···O_5_)is stronger than the interaction of hydrogen bond (O_3_-H_4_···O_5_). In brief, the stronger hydrogen bonding interaction is, the more prone ESIPT process is to occur.

### Computational details

With regard to our work, we have accomplished the theoretical calculationfor all parametersbased on the DFT and TDDFT methods by the Gaussian 09 program suite^42^. The TDDFT method has been extensivelyapplied toinvestigate the hydrogen bond dynamics in the S_1_ state^5^,^6^,^7^,^8^,^9^,^10^,^11^,^12^,^43^,^44^,^45^,^46^,^47^,^48^,^49^,^50^,^51^,^52^,^53^,^54^. Herein, the Becke One Parameter Hybrid Functionals (B1B95) has been used^54^. Moreover, the Pople’s 6–31++G (d, p) triple-ζ quality basis set with diffused and polarization functions has been carried out throughout^55^. The vibrational frequencies of the different configurations have been calculated to confirmreal local minimum of each optimized structurein S_0_ and S_1_ state. To simulate the solvent effect of quercetin in dichloromethane (DCM), we have selected the self-consistent reaction field (SCRF) method with the conductor-like screening model (COSMO) as the solvent model in our all calculations^56^. In this study, we have scanned the potential energy curves in the S_0_ andS_1_ state by means of increasing O_1_-H_2_ and O_3_-H_4_ bond lengthsat a fixed step size^57^,^58^,^59^. Therefore, the thermodynamic corrections of corresponding electronic states have been obtained via analyzingthe constrained optimization and vibrational frequency. Because we have employed the diffused functions, the calculation of vertical excitation energy would be unreliable. Therefore, the self-consistent field (SCF) convergency threshold has been set to be 10^−8^ (default settings are 10^−4^). In addition, we apply the RDG function to investigate the weak interaction types via the Multiwfn software^60^.

## Additional Information

**How to cite this article**: Yang, Y. *et al.* Theoretical Study of the ESIPT Process for a New Natural Product Quercetin. *Sci. Rep.* **6**, 32152; doi: 10.1038/srep32152 (2016).

## Figures and Tables

**Figure 1:**
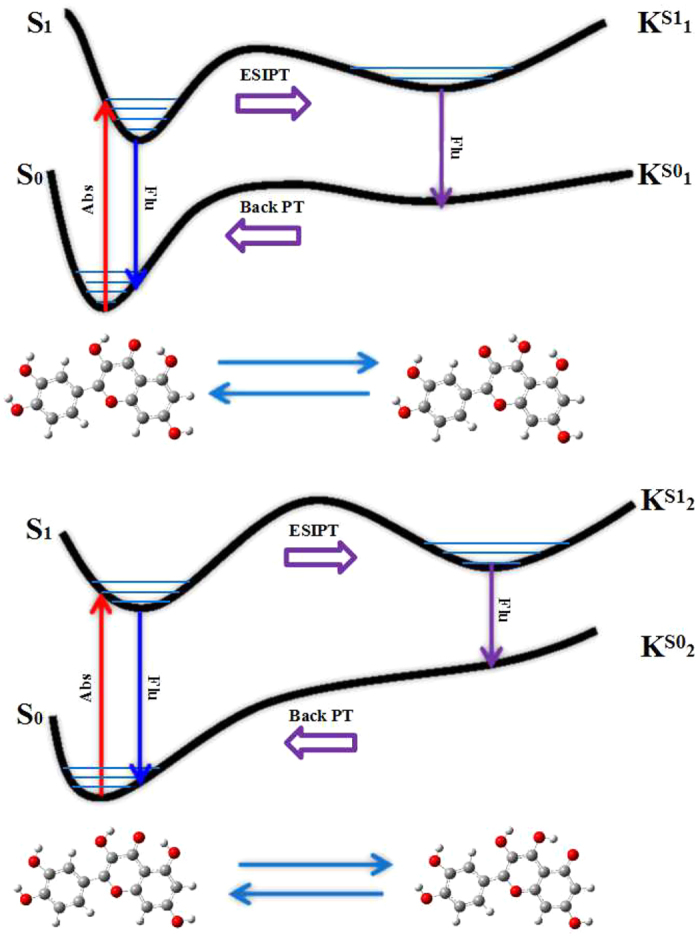
Two expected route graphs of the ESIPT process.

**Table 1:** The primary optimized bond lengths (Ǻ) and bond angles (°) of the hydrogen bond groups for the normal form and tautomeric forms (K_1_, K_2_) of the quercetin in the S_0_ and S_1_ state.

	Normal form		K_1_ form		K_2_ form	
	S_0_	S_1_	S_0_	S_1_	S_0_	S_1_
O_1_-H_2_	0.986	1.001	0.969	0.971	—	1.770
O_3_-H_4_	0.976	0.983	1.809	2.013	—	0.969
O_5_-H_2_	1.746	1.663	1.934	1.888	—	0.987
O_5_-H_4_	2.022	1.984	1.000	0.979	—	2.155
δ(O_1_-H_2_-O_5_)	148.2°	152.6°	140.5°	142.0°	—	146.5°
δ(O_3_-H_4_-O_5_)	117.9°	120.1°	124.0°	116.9°	—	113.1°

**Table 2:** The excitation wavelength (nm), the major composition of orbital transition with its ratio (%), and corresponding oscillator strengthen.

	Transition	λ (nm)	ƒ	Composition	CI (%)
quercetin	S_0_ → S_1_	371.69	0.6723	H → L	96.47%
	S_0_ → S_2_	320.13	0.0535	H−1 → L	94.58%
